# Comparative Study on the Thermal-Aging Characteristics of Cellulose Insulation Polymer Immersed in New Three-Element Mixed Oil and Mineral Oil

**DOI:** 10.3390/polym11081292

**Published:** 2019-08-02

**Authors:** Dawei Feng, Jian Hao, Ruijin Liao, Xin Chen, Lin Cheng, Mengna Liu

**Affiliations:** 1 State Key Laboratory of Power Transmission Equipment & System Security and New Technology, Chongqing University, Chongqing 400044, China; 2 Nanjing NARI Group Corporation, State Grid Electric Power Research Institute, Nanjing 211000, China; 3 Wuhan NARI Co. Ltd., State Grid Electric Power Research Institute, Wuhan 430077, China

**Keywords:** cellulose paper, polymer, mixed insulation oil, aging life, oil replacement, transformers

## Abstract

Cellulose paper, whose main component is cellulose polymer, has been widely used in oil-immersed power transformer that gradually deteriorates during transformer operation. Thermal aging is the main degradation form for cellulose paper immersed in insulation oil (oil–paper insulation) in a transformer. One of the most challenging issues in oil–paper insulation is inhibiting the aging of cellulose paper and extending its life. In this work, a comparative study was conducted on the thermal-aging characteristics of cellulose paper immersed in a novel three-element mixed insulation oil and mineral oil at 130 °C for 150 days. The key parameters of cellulose paper were analysed, including the degree of polymerization (DP), thermal-aging rate, surface colour, and AC breakdown voltage. The furfural content and acidity of the oil, as well as the AC breakdown voltage of the insulation oil were also analysed. The results show that the cellulose paper immersed in novel three-element mixed insulation oil had much higher DP values than that immersed in mineral oil after the same thermal-aging time. The mixed insulation oil could significantly inhibit the thermal aging of cellulose paper and prolong its life. The thermal-aging rate of the cellulose insulation polymer immersed in mixed insulation oil is significantly lower than that immersed in mineral oil, whether in the process of oil–paper insulation continuous aging or in the process of aging after oil replacement with unused insulation oil. The furfural generated by cellulose degradation in the novel three-element mixed insulation oil was also less than that in the mineral oil. The mixed insulation oil had a higher acidity value during the thermal-aging process, which was mainly due to the natural esters in the components of the mixed insulation oil. However, the AC breakdown voltage of the mixed insulation oil was always higher than that of the mineral oil. This study offers a new perspective in inhibiting the thermal aging of cellulose polymer in insulation oil.

## 1. Introduction

Polymers are widely used in electrical equipment, in which cellulose paper is the most important part of the main insulation in oil-immersed transformers. With the operation of transformers, cellulose paper gradually ages, and its mechanical and insulation strength decline [1], which may result in insulation failure. Most transformer failures are caused by insulation failures; thus, cellulose paper, which determines the insulation life of transformers as it is difficult to replace or repair throughout its lifetime [2], also determines the lifespan of transformers.

The degradation of cellulose paper in transformers is mainly caused by thermal aging. However, the thermal aging for cellulose paper in transformers is inevitable and irreversible, so scholars are sparing no effort to find ways to reduce the thermal-aging rate, thereby improving the life of transformers. For transformers with the same price, a long service lifespan indicates an average low cost, which is a considerable advantage on the cost of the entire power grid, because transformers are not only expensive but also extensive.

The following two methods have been proposed to decrease the aging rate of cellulose paper. The first is to change the properties of cellulose paper. For example, the aging rate of thermally upgraded cellulose paper is significantly lower than that of Kraft cellulose paper in the aging process, which has been successfully applied to transformers [3]. The second is using natural esters or mixed oil containing natural esters that impregnate cellulose paper to retard the aging rate of the cellulose polymer, which has been confirmed by many researchers [4,5].

However, the aforementioned methods can only be used on new transformers. On the one hand, replacing cellulose paper with thermally upgraded paper in old transformers is difficult. On the other hand, natural esters cannot be used to directly replace mineral oil because these insulating liquids cannot satisfy the IEC standard for mineral oil, which can only be used in redesigned transformers for safety.

To date, many transformers in power grids have been used for 20–30 years. In order to delay the aging rate of cellulose paper in these old mineral-oil transformers, mixed insulation oil containing natural esters has been further studied, and a new three-element mixed insulation oil was successfully developed at the end of 2017 [6]. The three-element mixed insulation oil contains 24% vegetable oil, and its main parameters can satisfy the IEC 60296:2012 for mineral oil. This finding indicates that the mixed insulation oil could be directly used to replace mineral oil in field transformers. More importantly, previous studies have shown that vegetable oil can retard the aging rate of cellulose paper [7]. Thus, the following aspects of the new mixed oil should be further confirmed: whether the mixed oil is able to delay the aging rate of cellulose paper and its delaying extent; and whether the mixed oil can delay the further aging rate of aged cellulose paper in a mineral oil–paper system after oil replacement with the mixed oil.

An accelerated thermal-aging test at 130 °C was conducted in the current study to clarify the aforementioned issues. In addition to the normal thermal aging of cellulose paper impregnated by mixed and mineral oil, oil replacement was conducted using unused mixed oil and mineral oil on mineral oil-immersed samples in the middle of the aging period. Parameters such as the degree of polymerization (DP) values of cellulose paper and the furfural content in oil were measured to study the thermal-aging characteristics of cellulose paper immersed in new three-element mixed oil and mineral oil.

## 2. Experiments

### 2.1. Materials and Sample Preparation

The cellulose paper and pressboard utilized in this experiment are listed in Table 1, and some copper bars were added in the oil–paper system to simulate the transformer environment. All these solid materials were provided by Yadongya Transformer Co., Chongqing, China. The main object for analysis in the current study is cellulose paper, which consists of 90% cellulose polymers, 6–7% hemicellulose, and 3–4% lignin [8]. The two insulation oils used were the three-element mixed insulation oil and Karamay 25# naphthenic mineral oil, and their main parameters are listed in Table 2.

### 2.2. Experiment Process

Both insulation oils were dried at 90 °C/50 Pa for 48 h to remove the dissolved gas and most of the moisture, and the moisture contents of the mixed oil and mineral oil after drying were approximately 14 and 10 ppm, respectively. The cellulose paper and pressboard were also dried at 90 °C/50 Pa for 48 h to maintain their moisture contents below 1%.

A total of 36 glass bottles were prepared for this thermal-aging experiment, and these bottles were divided into 4 groups, as shown in Table 3. Initially, 11 bottles were filled with 1 L of the dried mixed oil (Group B), and the remaining bottles were filled with 1 L of the dried mineral oil. The mass ratio of the insulation oil, cellulose paper, and cellulose pressboard was controlled to be 20:1:1. Thus, the corresponding mass of the dried cellulose paper and pressboard was added in each bottle. A copper bar equal to the mass of the cellulose paper was added to each bottle to simulate the transformers condition. Thereafter, these bottles were placed in 60 °C/50 Pa for 24 h to complete the oil-impregnation process. Then, the bottles were sealed under a nitrogen atmosphere and placed in a 130 °C thermostat.

Four hours later, two bottles, one from each group (A and B), were separately taken out as initial samples. Thereafter, samples in Groups A and B were periodically obtained based on previous experience. On the 20th day of aging, the DP values of cellulose paper dropped to approximately 600–700. The aged mineral oil in Groups C and D was then replaced with unused mineral and mixed oil, respectively. Subsequently, all samples in these four groups were placed at 60 °C/50 Pa for 24 h. These samples were then sealed in a nitrogen atmosphere. Finally, the four group samples were returned to the 130 °C thermostat for continued aging and periodically sampled for parameter measurement.

### 2.3. Parameter Measurements

The main parameters, such as DP values, breakdown voltage, and furfural content in oil, were measured during the aging process to analyse the aging characteristics of cellulose paper. Previous works indicate that there is a direct correlation between the DP of cellulose paper and its mechanical strength [9,10], and the mechanical strength of cellulose paper is very important in the power industry, thus the DP values are regarded as the most direct parameter to mirror aging extent of cellulose paper [11,12]. The tested parameters and their standards are listed in Table 4. For each sample, breakdown voltage was measured nine times, the other parameters were tested three times, and the average value was obtained as the final result.

The DP value of cellulose paper is the most important parameter in the current study. Thus, the measurement process is outlined in detail. According to IEC 60450-aml, the specific steps of the DP value test can be described as follows: first, the cellulose-paper sample is degreased with normal hexane; then, the cellulose paper is cut into pieces, and a certain mass of cellulose paper is weighted in accordance with the standard; next, the cellulose-paper fragments are placed into the cupriethy lenediamine solution to dissolve, and a vibration gauge is used to promote dissolution; finally, the viscosity of the mixed solution is measured by viscometer, and the DP values are calculated using the measurement results [13]. It should be noted that the DP value tested according to the standard is the viscosity average molecular weight of cellulose paper, which is mainly determined by macromolecules in cellulose paper and able to accurately mirror the mechanical properties of cellulose paper.

## 3. Results

### 3.1. DP Values of Cellulose Paper

**Figure 1:**
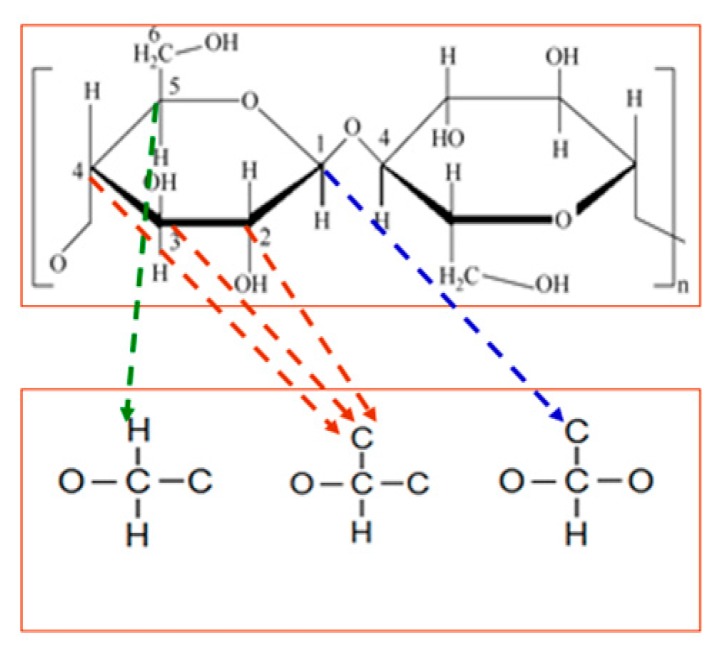
Chemical structure of cellulose insulation polymer.

**Figure 2:**
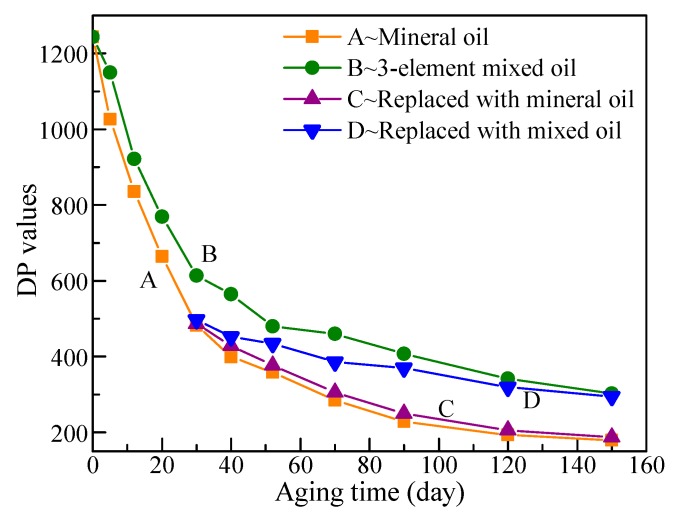
Test results of degree of polymerization (DP) values during the aging process.

Figure 1 illustrates the chemical structure of cellulose polymers. The cellulose consists of linear, polymeric chains of cyclic, β-D-glucopyranose units that are composed of C, H, and O elements [14]. Cellulose paper mainly comprises cellulose, and the DP value of cellulose is the average amount of glucose for the cellulose chain. Existing works have shown that DP values are closely related to the condition of the cellulose paper, such as mechanical strength and tenacity [9]. Thus, DP values are used to mirror the aging extent of cellulose paper, and the DP values of the cellulose paper in Groups A–D were tested and are shown in Figure 2.

The DP values of the cellulose paper rapidly decline in the initial aging period, and then decrease at a low rate due to the structure of cellulose paper. The cellulose paper can be divided into crystalline and amorphous zones in a microcosm, and the two types of zones are alternately joined to form cellulose paper. The molecular arrangement in amorphous areas is loose and disorderly compared with that in crystalline zones. Thus, amorphous areas are more likely to be broken due to the effects of heat, oxygen, and moisture [4]. Given that the crystalline and the amorphous areas generally alternate, the destruction of the amorphous areas leads to a rapid decrease in the DP values of the cellulose paper. However, the performance of the crystallization areas is relatively stable. Thus, the decrease rate of the DP values slows down in the later aging period.

The comparison between Groups A and B shows that the decreasing rate of DP values in Group B is apparently slower than that in Group A. This phenomenon confirms that the three-element mixed insulation oil can retard the aging rate of cellulose paper. The retardation effect was evident from the beginning of the aging experiment. On the 90th day, the DP values of cellulose paper impregnated by mineral oil dropped to 228, whereas those of cellulose paper impregnated by three-element mixed insulation oil remained above 400.

Groups C and D had oil replacement, and the cellulose paper exhibited different aging trends due to the different oils used. The aging rate of the cellulose paper was almost unchanged after oil replacement with unused mineral oil, and the DP values of cellulose paper in Group C almost coincided with those in Group A. By contrast, the aging rate of cellulose paper was apparently reduced after oil replacement with the three-element mixed insulation oil, and the DP values of the cellulose paper in Group D were considerably higher than those in Groups A and C. When the DP values of the cellulose paper in Group A dropped to 228 on the 90th day, those of the cellulose paper in Group D were 370. The comparison of the aging experiments shows that the retardation ability of mixed oil in terms of the aging rate of the cellulose paper is not only useful for new cellulose papers, but also effective for aged cellulose paper whose further aging rate is delayed.

The mixed oil delayed the aging rate of the cellulose paper due to the vegetable oils in the three-element mixed insulation oil. Vegetable oil comprises polar triglycerides; thus, mixed oil can hold additional water, and saturated-water content is 3–4 times that of mineral oil [6]. Based on the moisture equilibrium between oil and cellulose paper [15], some moisture in paper diffuses into insulation oil, reducing the moisture content in cellulose paper. Simultaneously, ester bonds of vegetable oil may hydrolyse [16], thereby consuming water and decreasing moisture content. Although this process generates macromolecular acids, they slightly affect the aging rate of cellulose paper [17]. Moisture is the number-one enemy of cellulose polymer aging [4], and the retardation of the aging rate of cellulose paper by mixed oil can be attributed to the reduction in moisture content in the cellulose polymer.

**Figure 3:**
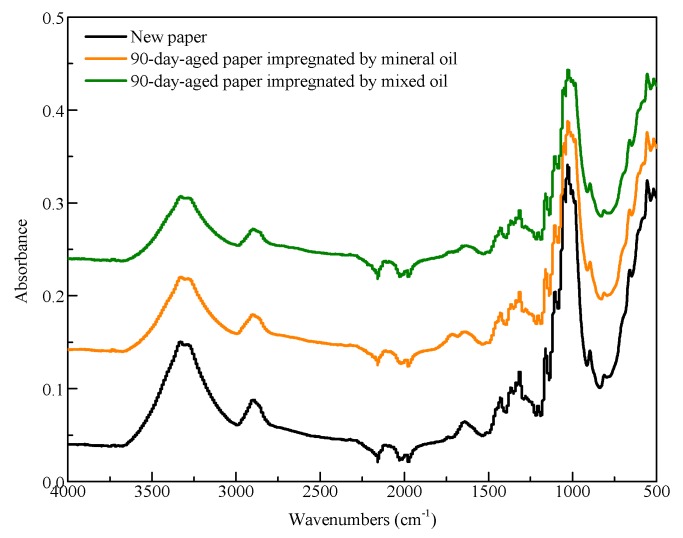
Attenuated total reflectance Fourier-transform infrared spectrometry (ATR-FTIR) of new paper and 90-day-aged cellulose paper.

Infrared spectrometry is always used to qualitatively analyse changes in molecular functional groups. Attenuated total reflectance Fourier-transform infrared spectrometry (ATR-FTIR) of new paper and 90-day-aged cellulose paper was measured, and the results are shown in Figure 3. It is worth noting that cellulose paper needs to be degreased with n-hexane before an ATR-FTIR test to avoid the influence of insulation oil. Figure 3 shows that the infrared spectrum of the cellulose paper in Group B was the same as that in the new cellulose paper and that in Group A, although some peak areas were different, which was due to the different aging extents of cellulose paper. For cellulose papers with different aging extents, the destruction extents of a chemical bond are different, thus exhibiting different characteristic peak areas. There was no new characteristic peak in Group B, indicating that no new chemical bond was massively produced. This phenomenon supports that the mixed oil retards the aging rate of cellulose paper by decreasing the moisture content in cellulose paper.

A zero-order kinetic equation (Equation (1)) is usually adapted to fit the variation of DP values during the aging process of cellulose paper to analyse the aging rate of cellulose paper [18]. Here is a simple algorithm to understand Equation (1). Cellulose paper is mainly made of cellulose, whose molecular formula is (C_6_H_10_O_5_)_n_. Understandably, the number of chain scissions is approximately equal to the increased number of cellulose chains because each breakage of a cellulose chain means that the number of molecules increases by one. Supposing m is the mass of the used cellulose paper, the number of chain scissions (N) during aging time 0–t can be calculated, as shown in Equation (2), in which 162 is the molar mass of the cellulose monosaccharide. Equation (3) is a variant of Equation (2), which suggests that 1/DP_t_−1/DP_0_ is a multiple of the number of chain scissions. Comparing Equations (1) and (3), k_0_ represents the relative chain-scission rate of cellulose over time t, as shown in Equation (4), in which N/t is the real chain-scission rate. The chain scission of cellulosic polymers means the aging of the polymer; thus, k_0_ can also be considered as a relative aging rate.

**Figure 4:**
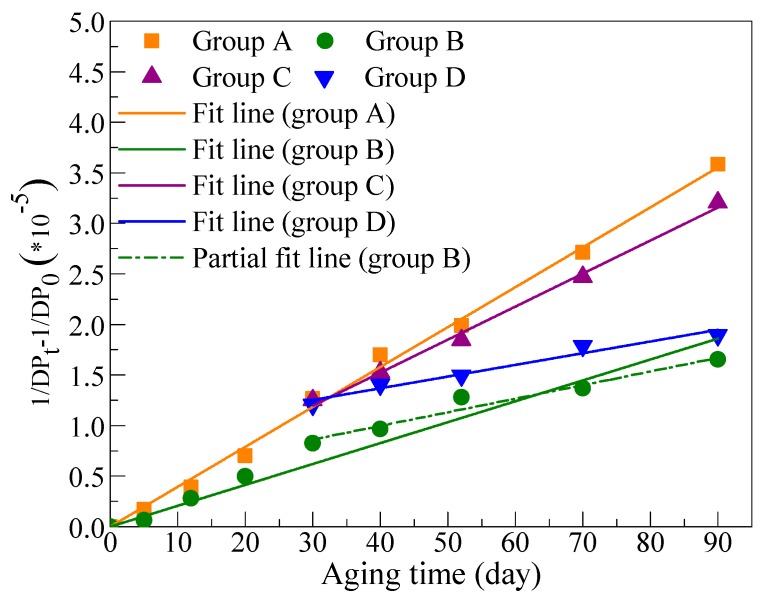
Test result of DP values during the aging process.

Here, 1/DP_t_−1/DP_0_ was calculated according to the data in Figure 2. The DP values of cellulose paper impregnated with mineral oil (Groups A and C) had fallen below 250 on the 90th day, so results before 90 days were fitted with a zero-order kinetic equation. For comparison, the DP values in Groups B and D conducted the same fitting, although there were still two points (120th and 150th day) not included; the fitting results are shown in Figure 4 and Table 5.

**Figure 5:**
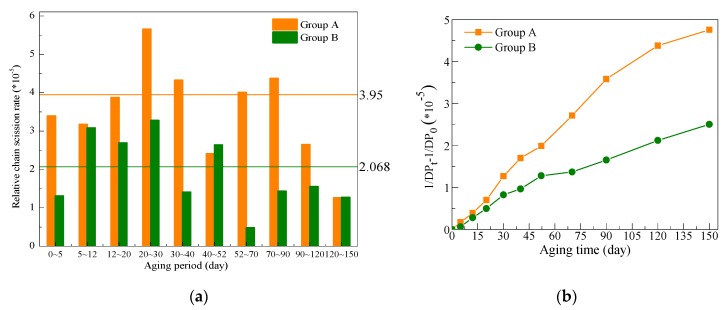
Relative chain-scission rate of cellulose in each aging period: (**a**) calculated k_t1~t2_; (**b**) slope of the family of two points for Groups A and B.

Group A showed good fitting effect with a zero-order kinetic equation before the 90th day, which was mainly because the relative chain-scission rate calculated at each aging period was around the average values (k_0_) during 0–90 days. Based on the above analysis, the relative chain-scission rate of cellulose in each aging period could be calculated with Equation (5), which evolved from Equation (1). The difference is that Equation (1) is for the entire aging process, while Equation (5) is for each aging period. The calculation results with Equation (5) for Groups A and B are presented in Figure 5a. In fact, the calculated results in Figure 5a are exactly the slope of the adjacent two points for Groups A and B, as shown in Figure 5b. The relative chain-scission rates of different aging periods in Group A fluctuated around the average value of 3.95 before 90 days, suggesting that the chain-scission rate was nearly the same. However, the chain-scission rate of cellulose paper impregnated with mineral oil decreased significantly after 90 days, which resulted from the leveling-off DP [19]. This indicates the chain-scission rate is very slow when DP value decreases to a low value, which is around 200 for cellulose paper impregnated by mineral oil [19]. For Group B (Figure 5a), however, not only was the chain-scission rate lower than that in Group A in each aging period, but the average chain-scission rate was also significantly decreased in the late aging period, especially for days 52–150, during which DP values were below 500. This result suggests that the mixed oil retarded the aging of the cellulose paper mainly at the late aging period, which can also be found in Figure 4.

(1) $$1/DP_{t}-1/DP_{0}=k_{0}\cdot t$$

(2) $$N=m/(162\times DP_{t})-m/(162\times DP_{0})$$

(3) $$1/DP_{t}-1/DP_{0}=\frac{162}{m}\times N$$

(4) $$k_{0}=\frac{162}{m}\times \frac{N}{t}$$

(5) $$1/DP_{t2}-1/DP_{t1}=k_{t1\sim t2}\cdot (t2-t1)$$

where *DP*_0_ denotes the initial DP value of cellulose paper; *t* is aging time (days); *DP_t_* is DP value at time *t*; *k*_0_ is a constant, which indicates the relative aging rate of cellulose paper in Equation (1); *N* is the number of chain scissions, in mol; *m* is the mass of the cellulose paper, in g; and 162 is the molar mass of the cellulose monosaccharide, in g/mol.

Insulation oil was replaced in Groups C and D, and data in the late aging period (30–90 days) were fitted with Equation (1), and fitting results were satisfactory (Figure 4). Similarly, results in Group D showed that the mixed oil can significantly delay the aging rate of cellulose paper in the late period.

Fitting results in Table 5 show that the aging rate of cellulose paper in Group B is apparently slower than that in Group A. This phenomenon confirms that the three-element mixed-insulation oil strongly retards the aging rate of cellulose paper. The oil in Group C was replaced with unused mineral oil, thereby decreasing the aging rate of cellulose paper to 0.83 times of that in Group A. The mineral oil in Group A underwent a long aging time and produced additional aging byproducts such as acid, which further accelerate the aging of the cellulose paper [17]. As a result, the cellulose paper in Group C slowly slightly degraded. The oil in Group D was replaced with three-element mixed-insulation oil, and the aging rate of cellulose paper decreased to approximately 0.29 times the aging rate of that in Group A, which shows the delaying effect of the mixed oil on the further aging rate of aged cellulose paper.

Cellulose paper loses most of its mechanical strength when DP values decrease below 250. Assuming that DP values drop to 250 as the life endpoint of cellulose paper, then the thermal-aging life of cellulose paper in Groups A and B could be calculated with Equation (6), which is a variation of Equation (1), and the results are listed in Table 6.

(6) $$t=\left(1/DP_{t}-1/DP_{0}\right)/k_{0}$$

Considering the change in aging rate in Groups C and D, the lifetime is divided into two parts. Thermal-aging life in Group C after the 30th day was calculated with the fitting results for Group C in Table 5, and then total aging life can be calculated, as shown in Table 6. For Group D, DP value only reduced to 294.4 after 150 days of aging; thus, its accurate aging life could not be obtained based on the experiment results. Similar to Group C, aging life after 30 days was estimated based on the fitting results in Table 5, and the result was 171.9 days; thus, the total lifespan of cellulose paper in Group D could be recorded as 201.9 days.

Comparing the results in Groups A and B, the aging life of cellulose paper was significantly improved by the mixed oil, which increased by 86.8% when compared with the cellulose paper in Group A. Supposing that mineral oil is replaced with mixed oil when the DP is approximately 600 (Group D), the aging life of cellulose paper is extended to 2.5 times. The retarding-aging effect of mixed oil is mainly reflected in the period of low DP values, especially those below 500, during which aging rate was considerably reduced. Therefore, oil replacement with mixed oil in the later aging stage could also delay the further aging rate of the aged cellulose paper.

An interesting phenomenon is that cellulose paper life in Group B was significantly lower than that in Group D, which was mainly due to the different methods used in the fitting. In fact, the aging rate of cellulose paper in Group B should have been near that in Group D in the late aging period due to the consistent action of the mixed oil, which was proved by that the slope of the partial linear fitting of Group B was close to that in Group D in the late aging period in Figure 4. Supposing the aging life of cellulose paper after the 90th day in Group B is roughly estimated using the slope of partial linear fitting result, the remaining life after 90 days would approximately be 114.4 days. This suggests that the total life of cellulose paper in Group B was 204.4 days, which is closed to the estimated lifespan in Group D. When oil replacement was conducted, the DP values of cellulose paper in Group B were higher than those in Group D. However, the oil in Group B underwent longer aging time, resulting in more byproducts that accelerate the aging rate of cellulose paper [17], so the final estimated life was nearly the same as that in Group D.

Someone may doubt the abovementioned estimation method for Groups B and D. In fact, after 90 days, there are two more points that were tested during the experiment, which could be used to verify the correctness of the estimation. The k_0_ used in the estimation in Groups B and D was 1.347 * 10^–5^ and 1.157 * 10^–5^, respectively. Assume k_0_ remains unchanged after 90 days, the DP values of cellulose paper in Groups B and D at 120 and 150 days could be calculated with Equation (7). Take the 120th day in Group B as an example: if t1 equals 120 and t2 equals 90, then Equation (8) can be obtained. The measured DP_90_ is 406.7, so the DP value on the 120th day can be calculated with k_0_ (1.347 * 10^–5^). All calculated results are shown in Table 7. The results show that the calculated results are very close to the measured DP values, which proves that partial fitting in Groups B and D can be used to predict the subsequent development trend of DP values.

(7) $$\begin{array}{l} 1/DP_{t1}-1/DP_{0}=k_{0}\cdot t1\\ 1/DP_{t2}-1/DP_{0}=k_{0}\cdot t2 \end{array}\}\Rightarrow 1/DP_{t1}-1/DP_{t2}=k_{0}\cdot (t1-t2)$$

(8) $$1/DP_{120}-1/DP_{90}=k_{0}\cdot (120-90)$$

where *t*1, *t*2, 90 and 120 denote aging time, in days.

Although lifespan estimation in Groups B and D is not absolutely precise, they showed apparently longer lifespan than that in Groups A and C, because the DP values of cellulose paper immersed in the mixed oil (Groups B and D) stayed around 300 at 150th day, which far exceeded the 81-day life of cellulose paper immersed in mineral oil (Group A). The results demonstrate that the three-element mixed insulation oil can retard the aging rate of cellulose paper, which is hopeful to replace aged mineral oil in old transformers to extend the aging life of cellulose paper in field transformers.

### 3.2. Colour and Breakdown Voltage of Cellulose Paper

**Figure 6:**
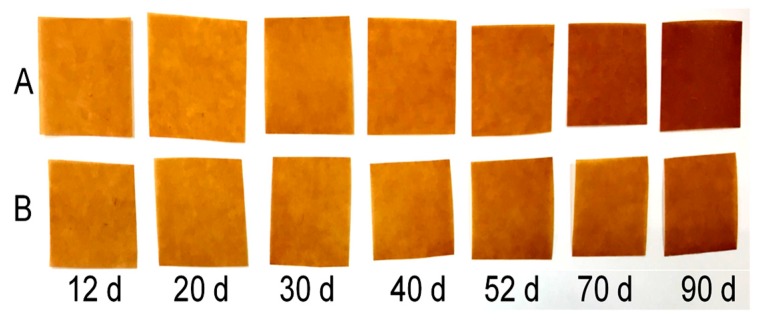
Colour of aged cellulose paper impregnated with mineral oil and mixed insulation oil: (**A**) cellulose paper impregnated with mineral oil; (**B**) cellulose paper impregnated with mixed oil.

Figure 6 shows the colour variation of cellulose paper in Groups A and B during the aging process. The colour change of the cellulose paper was not apparent in the initial and middle aging period, but obviously deepened in the late period of aging. Interestingly, the colour of the cellulose paper aged in three-element mixed insulation oil was significantly lighter than that aged in mineral oil after being aged for 52 days, which is consistent with the aging degree of cellulose paper in different groups. The dark colour of the cellulose paper reflected its serious aging, namely, the cellulose paper with a dark colour had lower DP values; thus, the aging extent of cellulose paper in transformers could roughly be compared by observing the colour. This result shows that mixed oil retards the aging of cellulose paper in appearance.

**Figure 7:**
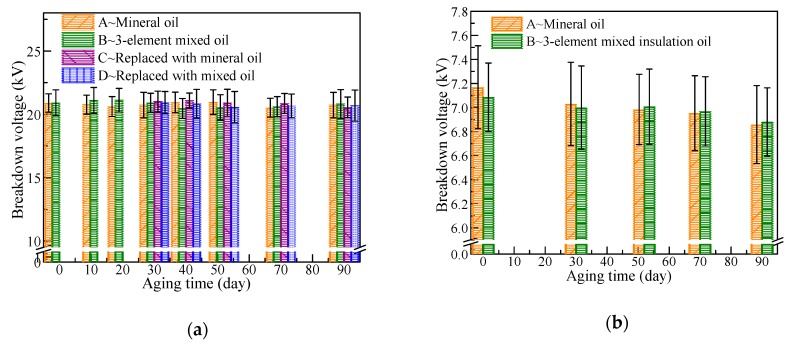
Breakdown voltage of cellulose polymers impregnated with different oils: (**a**) cellulose pressboard; (**b**) cellulose paper.

Breakdown strength is an important parameter for cellulosic paper in power-equipment applications. Low breakdown strength may easily cause insulation failure, thus destroying the power equipment. As shown in Figure 7a, the breakdown voltages of cellulose pressboards impregnated with two insulation oils did not demonstrate apparent changes throughout the aging process, including the group after oil replacement.

In comparison, the breakdown voltage of cellulose paper (Figure 7b) gradually decreases with aging time, and mineral-oil-impregnated cellulose paper shows remarkable decline. In the middle and late aging period, the average breakdown voltage of the mixed-oil-impregnated cellulose paper is superior to that of the mineral-oil-impregnated paper. During the aging process of cellulose paper, the internal molecule structure is destroyed and defects occur, which is easily broken down with the effect of an electric field [20]. The three-element mixed insulation oil can effectively delay the aging rate of cellulose paper. Therefore, cellulose paper immersed by mixed oil is less aged, which is the main reason for the mixed-oil-impregnated paper having high breakdown voltage in the middle and late periods of aging.

## 4. Mixed-Oil Performance

### 4.1. ATR-FTIR Spectra of the Mixed Oil

**Figure 8:**
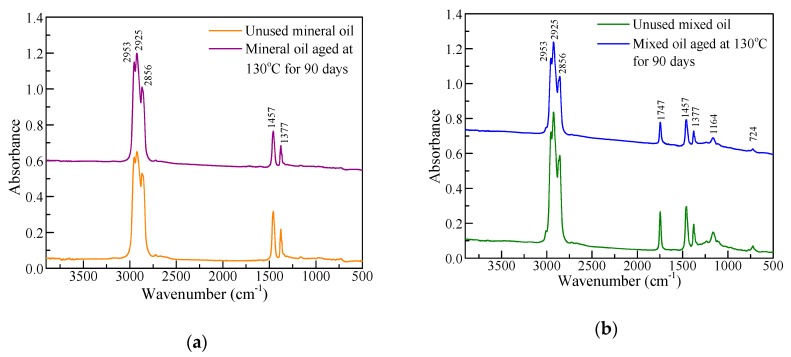
ATR-FTIR spectra of unused and aged insulation oils: (**a**) mineral oil; (**b**) three-element mixed oil.

Figure 8 presents the ATR-FTIR of unused and 90-day-aged insulation oils in which the curves of 90-day-aged insulation oils were shifted up by 0.5 for clarity. The chemical groups corresponding to the absorption peaks in infrared spectra are shown in Table 8 [21]. Since the mineral oil accounted for 76% in the mixed insulation oil, the characteristic peaks in Figure 8a are also apparent in Figure 8b, which shows that the functional groups of the mixed oil had not changed during the aging process, indicating that the global property of the mixed oil remained the same during the entire aging process. For both insulation oils, a decrease in the area of some absorption peaks suggests that functional groups such as –CH_2_– at 2925 cm^−1^ and C–O at 1724 cm^−1^ were partially destroyed. The overall increase in the absorbance of aged oil is due to the colour-deepening of the insulation oil during aging, which was mainly caused by the oxidation of insulation oil [22].

### 4.2. Furfural Content in Oil

**Figure 9:**
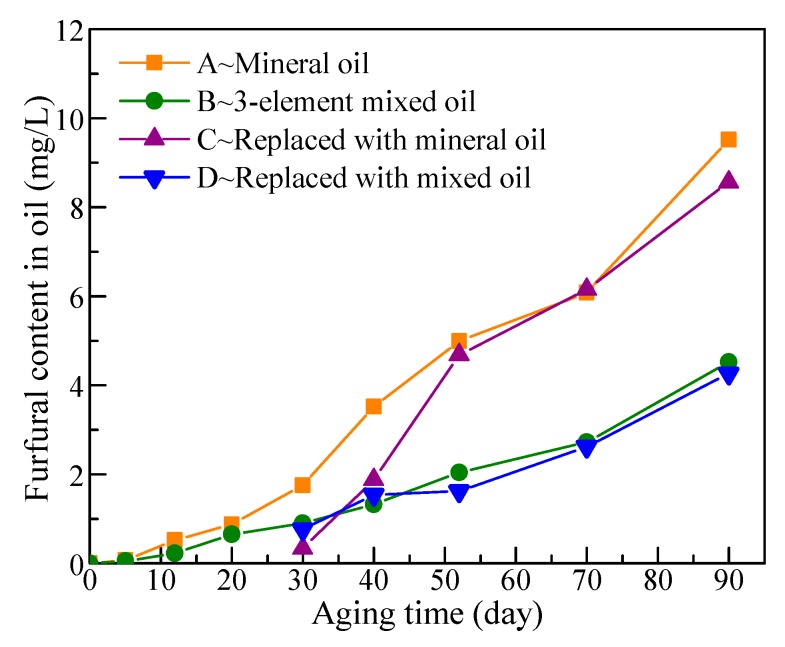
Furfural content in oil during aging process.

Furfural is a byproduct during the degradation process of cellulose paper, and furfural content in oil is regarded as an effective parameter reflecting the aging state of cellulose paper [23]. Figure 9 shows that the furfural content in both oils gradually increased with aging time due to the continuous degradation of cellulose paper. Furfural content in mixed oil was apparently lower than that in mineral oil, which was nearly only half the concentration of that in mineral oil on the 90th day. For the mineral oil in Group A, furfural content was approximately 5 mg/L when DP values of cellulose paper decreased to 360 on the 52nd day. Regarding mixed oil in Group B, furfural content was approximately 4.52 mg/L when DP values decreased to 400 on the 90th day. Comparing the two points, DP values in Group B were slightly high, while furfural content in oil was slightly lower than that in Group A; thus, the produced furfural amount did not much differ, assuming that the DP dropped to the same value. Therefore, mixed oil had low furfural content varying with time mainly due to the low aging rate of cellulose paper, which also showed the mixed oil retarding the aging rate of cellulose paper.

For Groups C and D, oil replacement did not remarkably affect furfural content in oil, which was only slightly lower than in groups without oil replacement in the subsequent aging process. This finding is mainly due to most of the furfural content existing in cellulose paper [13], and furfural content in oil was low when oil replacement was conducted. Thus, loss of furfural content was small during the oil-replacement process.

**Figure 10:**
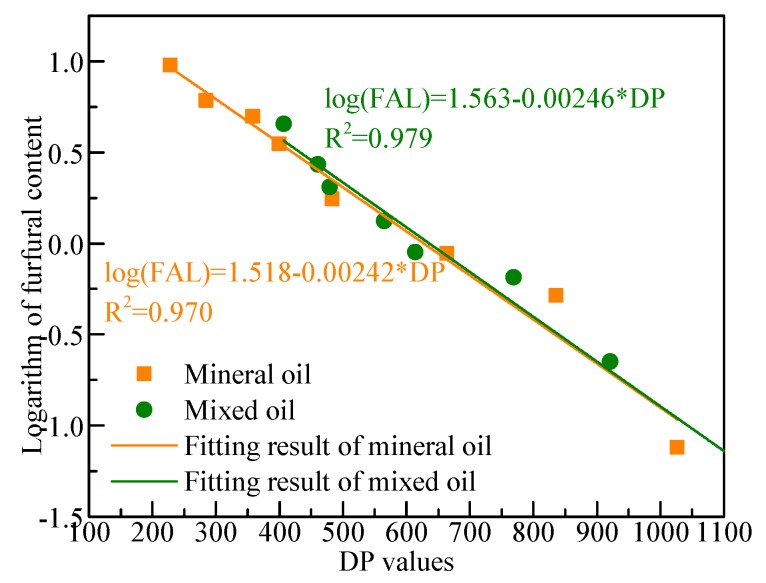
Relationship between furfural content in oil and DP values.

Previous results showed that there is a linear relationship between the logarithm of the furfural content in oil and the DP values of cellulose paper during the aging process. For the test results in Groups A and B, fitting was performed with Equation (9), and results are shown in Figure 10. The fitting results were nearly the same in intercept and slope, indicating that furfural content in oil was close for the two oils impregnating the cellulose paper when DP decreased to the same value. This also shows that the difference in furfural content in the two oils resulted from the difference in aging rate.

(9) log(FAL)=a+b×DP

where *FAL* denotes furfural content in oil; DP denotes the measured DP values of cellulose paper.

### 4.3. Acid Values of Insulation Oil

**Figure 11:**
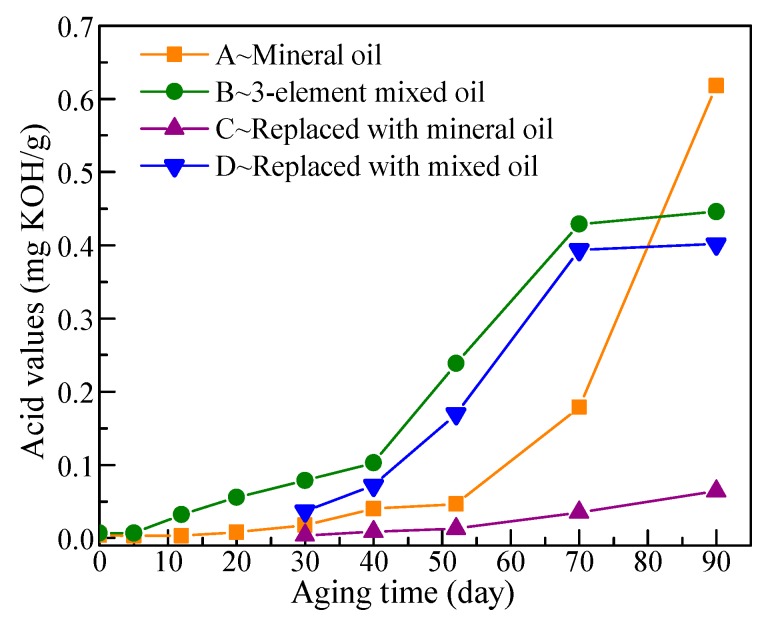
Acid values of insulation oils during the aging process.

The used indicator in the acidity test was alkali blue 6B, and the acid values of the mixed and mineral oil varying with aging time are shown in Figure 11. Mineral oil can maintain a low acid value in the initial and middle aging period, and a considerable increase in acid value in mineral oil only occurs in the later aging stages. Oil replacement with new mineral oil maintains the acid value of insulation oil at a low level. The acid value of mixed oil gradually increases from the beginning and is apparently larger than that of mineral oil. With the effect of oxygen and water, the vegetable oil in the mixed oil accelerates the production of acids [24], thereby increasing the acid values of the mixed oil. The acids produced from vegetable oil are basically macromolecular acids from the hydrolysis of esters [25], and these acids hardly affect the aging rate of cellulose paper [17]. At the end of the aging process, the increase rate in acid value of the mixed oil apparently decreased, which is consistent with the previous result obtained by M. M. Ariffin regarding natural esters [26]. For mineral oils, acid values are often used to represent the aging degree of insulation oil. High acid value not only indicates considerable reduction in the overall performance of insulation oil but also accelerates the aging rate of the cellulose paper. Therefore, the standard in China stipulates that the acid value of mineral oil in transformers should be less than 0.1 mg KOH/g. However, this requirement is demanding for mixed oils because their acid value is generally high, and the acid can hardly affect oil properties and the aging rate of cellulose paper; thus, different standards should be used for the acid values of mixed oil.

### 4.4. Breakdown Voltage of Oil

**Figure 12:**
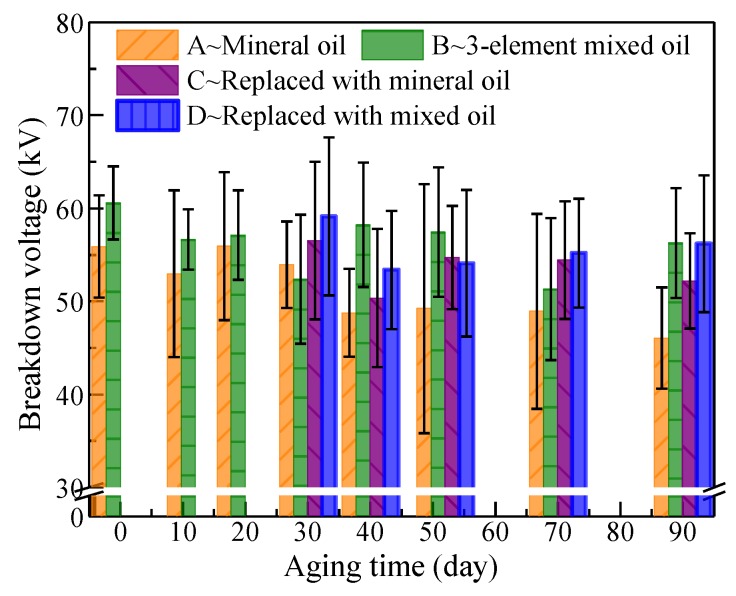
Breakdown voltage of insulation oil during the aging process.

Although breakdown voltages of oils are not directly related to polymer degradation, they were still measured for breakdown voltage indicating that dielectric strength is one of the most important parameters of insulation oil. Average breakdown voltages with error bars are shown in Figure 12. The breakdown voltages of the mixed and mineral oil only slightly decreased during the entire aging process, which is consistent with the previous results on mineral oil [27]. The aging of insulation oil can only slightly affect breakdown voltage, in which mineral oil is the most affected and only drops by approximately 17.8% in the entire aging process. For mineral oil in Group C after oil replacement, its breakdown voltage was basically equivalent to that of mixed oil because its aging time being shorter than that in Group A and the inherent error of breakdown measurement.

The three-element mixed insulation oil had higher breakdown voltage than that of mineral oil due to the high saturation-moisture content of mixed oil [6], and this advantage was manifested throughout the aging process. For insulation oils in Group D after oil replacement, the same trend was presented. This is good news for mixed oil, for it can not only delay the aging rate of cellulose polymers, but also maintain good insulation property at the same time, which is beneficial to its applications.

## 5. Conclusions

Previous works showed that unused three-element mixed oil, which can be directly used to replace mineral oil, possesses excellent performance [6]. This study mainly focused on the aging characteristics of a cellulose polymer impregnated with the mixed oil, with or without oil replacement. The three-element mixed oil exhibited good performance during the aging process, which is beneficial for field applications of the mixed oil. The main conclusions are listed as follows:

(1) The three-element mixed oil could delay the aging rate of cellulose paper, and this retardation was evident from the beginning. Supposing DP values drop to 250 at the end of service life, the estimated life of cellulose paper immersed with mixed oil can reach 2.5 times that of cellulose paper impregnated with mineral oil.
(2) For a mineral oil–paper system, oil replacement with three-element mixed insulation oil in the middle aging period can delay the further aging rate of the cellulose paper, and aging life may significantly increase. This result may be applied to old field transformers to increase their insulation life.
(3) Some other parameters, such as the light colour of the aged paper impregnated with the mixed oil, and the low furfural content in the mixed oil also reflect that mixed oil retards the aging of the cellulose paper. In addition, mixed oil maintains its good performance throughout the aging process, which is useful for applications of mixed oil in the power industry.

## Figures and Tables

**Table 1:** Parameters of cellulose polymers.

Material	Density (g/cm^3^)	Thickness (mm)	Provider
Cellulose paper	0.933	0.108	Yadongya Transformer Co., Chongqing, China
Cellulose pressboard	1.20	0.45	

**Table 2:** Parameters of the three-element mixed insulation oil and 25# mineral oil.

Property	Three-Element Mixed Insulation Oil	Mineral Oil
Appearance	Transparent, free water or suspended matter, yellow	Transparent, free water or suspended matter, colourless
Kinematic viscosity (40 °C, mm^2^/s)	11.65	9.2
Moisture content (ppm)	14	11
Breakdown voltage (2.5 mm, kV)	78.6	72
Density (20 °C, kg/m^3^)	886.8	890
tan δ (90 °C)	0.0047	0.001
Acid value (mg KOH/g)	0.007	0.004
Relative permittivity (90 °C)	2.32	2.12

**Table 3:** Sample grouping and oil treatment.

Group	A	B	C	D
Bottle number	11	11	7	7
Initial Oil	Mineral oil	Mixed oil	Mineral oil	Mineral oil
Oil replacement	No	No	Yes	Yes
Time for oil replacement	-	-	20th day	20th day
Oil replaced with	-	-	Mineral oil	Mixed oil

**Table 4:** Test method and standard of parameter measurement.

Parameter	Method	Standard	Equipment
DP value of cellulose paper	Pulp viscosity	IEC 60450	NCY viscosity meter
Breakdown voltage of cellulose paper	Unidiameter electrode	IEC 60243	Test transformer
Breakdown voltage of oil	Standard oil cup (2.5 mm)	IEC 60156	Test transformer
Acidity of oil	KOH solution titration	IEC 62021	-
Furfural content in oil	high performance liquid chromatography	IEC 61198	Agilent 1260
Infrared spectrometry	Attenuated total reflectance	-	Nicolet iS5

**Table 5:** Fitting results of DP values with kinetic equation.

Group	k_0_ (×10^−5^)	R-Square
A	3.95	0.998
B	2.068	0.978
C	3.268	0.994
D	1.157	0.951
Partial B	1.347	0.942

**Table 6:** Expected life of cellulose paper in thermal aging test (130 °C).

Group	Life after 30th Day	Total Life (d)
A	-	80.9
B	-	151.1
C	59.5	89.5
D	171.9	201.9

**Table 7:** Calculated and measured DP values after 90 days.

Group	Calculated DP Value		Measured DP Value		
	120th Day	150th Day	90th Day	120th Day	150th Day
B	349.3	306.1	406.7	341.8	302.3
D	322.0	284.9	370.1	319.5	294.4

**Table 8:** Chemical groups corresponding to absorption peaks.

Wavenumber (cm^–1^)	Chemical Group	Vibration Type
2953	–CH_3_	asymmetric stretching
2925	–CH_2_–	asymmetric stretching
2856	–CH_2_–	symmetric stretching
1747	C=O	stretching
1457	–C–CH_3_	asymmetric bending
1377	–C–CH_3_	symmetric bending
1164	C–O	stretching
724	–(CH_2_)n– (n >4)	in-plane bending
